# Asymptomatic Neurosyphilis in HIV infected patients at a Brazilian HIV and AIDS specialized service: a cross sectional study

**DOI:** 10.1590/0037-8682-0418-2021

**Published:** 2022-02-25

**Authors:** Cláudio Queniti Hirai, Deborah de Castro Moreira, Danielle Cristina Tita Granzotto, Eniuce Menezes de Souza, Jorge Juarez Vieira Teixeira, Dennis Armando Bertolini

**Affiliations:** 1 Universidade Estadual de Maringá, Programa de Pós-Graduação em Ciências da Saúde, Maringá , PR, Brasil.; 2 Universidade Estadual de Maringá, Departamento de Estatística, Maringá, PR, Brasil.; 3 Universidade Estadual de Maringá, Departamento de Análises Clínicas e Biomedicina, Maringá, PR, Brasil.

**Keywords:** HIV, Acquired Immunodeficiency Syndrome, Neurosyphilis, Co-infection, Syphilis

## Abstract

**Background:**

Many human immunodeficiency virus (HIV) and syphilis co-infected patients are not diagnosed, which may evolve into asymptomatic neurosyphilis (ANS). We studied the occurrence of ANS an HIV-infected population.

**Methods:**

This was a cross-sectional study of cerebrospinal fluid (CSF) samples collected from patients co-infected with HIV and *Treponema pallidum*. Social-demographic and clinical-laboratory characteristics were studied.

**Results:**

Of the 348 patients infected with HIV, 33 (9.5%) had reagent treponemic and non-treponemic tests. CSF was collected from 19 asymptomatic patients. Of these, 8 (42.1%) presented with laboratory alterations suggestive of ANS.

**Conclusion:**

Social-demographic and clinical-laboratory variables should be considered for the indication of CSF collection.

Globally, more than one million sexually transmitted infections (STIs) occur daily^1^. The prevailing STIs are chlamydia, gonorrhea, trichomoniasis, and syphilis, corresponding to 33.8%, 23.1%, 41.5%, and 1.7%, respectively^1^. Despite its low percentage, when compared to other STIs, syphilis can evolve into chronic and asymptomatic forms, leading to an expansion of its propagation. It is worth mentioning that syphilis infection greatly increases the transmission and acquisition of HIV^1^. 

Syphilis is the most common STI among patients with HIV infection^2^, with a median of 9.5% in developed and developing countries^3^. Studies have shown different prevalence rates of asymptomatic neurosyphilis (ANS) in patients co-infected with HIV and syphilis. Rotman *et al.* retrospectively studied 230 patients with cerebrospinal fluid (CSF) analysis for 10 years in American hospitals and found 70 (8.57%) ANS cases^4^.

Lumbar puncture is recommended in patients with clinical evidence of neurological involvement, such as cognitive dysfunction, motor or sensory deficits, ophthalmological or auditory symptoms, cranial nerve palsy and meningitis or stroke signs or symptoms, tertiary syphilis with cardiovascular or gum disease signs or symptoms, and in cases of treatment failure^5^ ^-^ ^7^. CSF abnormalities are often associated with a CD4 + T lymphocyte count (CD4TL) ≤ 350 cells/mm³ and/or a Venereal Disease Research Laboratory (VDRL) titer ≥ 1:32^5^ ^,^ ^8^. 

Polymerase chain reaction (PCR) and dark field microscopy are the gold standard techniques for mucocutaneous lesions; however, in CSF, they have low sensitivity when compared to serological assays. Thus, CSF serological assays have been the mainstay of diagnosisfor neurosyphilis. In CSF, VDRL has good specificity but limited sensitivity; however, it is still considered the gold standard assay^9^. A reactive CSF-VDRL confirmed the diagnosis of neurosyphilis, but a negative test does not exclude it^10^.

In Brazil, as neurosyphilis has not been reported, there are few epidemiological data, especially on its asymptomatic form. The aim of this study was to analyze the frequency and characteristics of ANS in an HIV-infected population, followed by an HIV/AIDS reference clinic.

We conducted across-sectional study with consecutive sampling according to the following inclusion criteria: patients HIV-infected with serological treponemic and non-treponemic tests reagent for syphilis; fully asymptomatic; older than 16 years; not pregnant; with recent viral load and CD4LT count examinations; without diseases that justified liquor alterations, without neurological, ophthalmological, otological, or tertiary syphilis signs or symptoms; increase or maintenance of VDRL titers, compared to the previous syphilis exam; latent syphilis independent of previous treatment for syphilis; and CD4LT count and/or VDRL serological titer.

People living with HIV/AIDS (PLHIV) in this study were clinically attended between October 1, 2018 and September 30, 2019 in the Specialized HIV and AIDS Assistance Service (SAE) of the Inter-municipalities Health Consortium of the North of Paraná, a reference clinic for 21 municipalities, with 224,721 habitants, located in the city of Cornélio Procópio, State of Paraná. Selected patients underwent CSF collection at Cornélio Procópio’s House of Mercy Hospital. Blood and CSF examinations were performed in the reference laboratories of the Brazilian Unified Health System.

The following liquor characteristics were considered as neurosyphilis: VDRL at any title, or/and leukocyte count (pleocytosis) higher than 20 cells/mm^3^, and/or a protein increase over 40 mg/dL. A questionnaire was administered to patients for clinical-epidemiological and social-demographical data collection. Patient date on previous diagnoses, treatment, and follow-up were gathered from clinical records. The following definitions were applied: (a) adequate treatment, patients with treatment and laboratory monitoring; (b) inadequate treatment, patients with inadequate treatment or laboratory monitoring; and (c) lack of syphilis treatment, patients without previous treatment history.

The frequency distribution of the clinical-epidemiological and social-demographic variables were analyzed. Non-parametric statistical tests were applied, such as the chi-square test of independence. Statistical analysis was conducted using R software, version 3.5.3. The Ethics Committee of the State University of Maringá approved the project (CAAE 13174419.3.0000.0104; Protocol no. 3.390.519), in compliance with the guidelines and regulatory requirements for human research. All participants who agreed to participate signed an informed consent form.

During the study period, from the 393 patients with HIV infection attended by SAE, 348 were tested for syphilis, of which 73 (21%) presented treponemic test reagents to syphilis. Of these, 31 (42.5%) were considered cured after adequate treatment and a follow-up with negative VDRL, 9 (12.3%) lost clinical follow-up, and 33 (45.2%) had treponemic and non-treponemic test reagents for syphilis. From the 33 patients, target of this study, 19 (57.6%) patients collected the CSF sample (Figure 1). The majority were male (73.7%), white (52.6%), single (52.6%), and aged between 20 and 49 years (73.7%).

**FIGURE 1:**
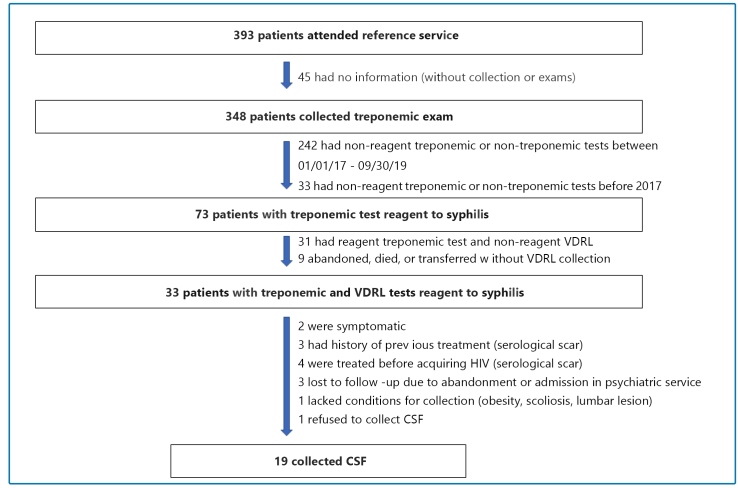
Patient selection flowchart. VDRL: Veneral Disease Research Laboratory; **HIV:** human immunodeficiency virus; CSF: cerebrospinal fluid.

Retrospective analysis of medical records of the 19 patients (Supplementary Table 1) indicated that 15 (78.9%) had a previous history of syphilis treatment, of which 8 (53.3%) had serum VDRL titers ≥1:32, and 9 (60%) with an CD4LT count lower than 350 cells/mm^3^ at the time of their diagnosis, which could suggest CSF collection^7^ ^-^ ^9^. However, they did not undergo such procedures. By analyzing previous treatments, some conditional probabilities were estimated. Considering the previous treatment scheme, 43,8% of the patients who underwent an inadequate previous treatment developed ANS. 

Neurosyphilis treatment must be conducted with crystalline penicillin G, since benzathine penicillin G does not achieve therapeutic levels in CSF^6^ ^,^ ^7^. Considering that CSF collection was conducted in 14 patients (93.3%) only a year after diagnosis, the lack of past information in the medical records hampered the correct interpretation of the patients’ clinical situation. We wish to note that, during the previous syphilis treatment, none of the patients collected CSF to assess neurosyphilis.

Of the 19 patients that collected CSF, 4 (21.1%) presented detectable viral load values over 40 copies/mL in plasma at the time of CSF collection, suggesting irregular ART use or efficacy failure. Five patients (26.3%) had an CD4LT count < 350 cells/mm^3^. Two patients (10.5%) showed VDRL reagent in blood with titers ≥1:32 (Table 1). CSF examination revealed altered results in eight patients (42.1%). The individual characteristics of the 19 patients who collected CSF are detailed in Supplementary Table 1. Patient 7 was clinically evaluated and had no history, signs, or symptoms of current disease or history of tuberculosis.

**TABLE 1:** Clinical-laboratorial characteristics of the 19 patients with HIV/syphilis co-infection, in Cornélio Procópio, State of Paraná, between 10/01/2018 and 09/30/2019.

Variables	Patients		P-value
	n	%	
**Time of HIV diagnosis**			0.490*
< 5 years	9	47.4	
≥ 5 years	10	52.6	
**ART use**			<0.001
Yes	18	94.7	
No	1	5.3	
**Viral Load (copies/mL)**			0.196*
≥ 40	4	21.1	
< LD	15	79.9	
**CD4LT count (cells/mm** ^3^ **)**			0.251*
< 350	5	26.3	
≥ 350	14	73.7	
**VDRL (serum)**			<0.001
<1:32	17	89.5	
≥ 1:32	2	10.5	
**VDRL (CSF)**			<0.001
NR	18	94.7	
R	1	5.3	
**Leukocytes (CSF - cells/mm** ^3^)			<0.001
< 20	17	89.5	
≥ 20	2	10.5	
**Proteins (CSF - mg/dL)**			0.346*
< 40	12	63.2	
≥ 40	7	36.8	

*Not significant, at a 5% level of significance. ART: antiretroviral therapy; CD4LT: CD4+ T lymphocytes; VDRL: Veneral Disease Research Laboratory; CSF: cerebrospinal fluid; ND: not detectable; LD: lower than the minimum detection limit; NR: not reagent; R: reagent.

Neurosyphilis can occur at any phase of syphilis, and individuals inadequately treated, especially individuals with co-infection, present less therapeutic efficacy, allowing neurological impairment^11^. *T. pallidum* invasion of the central nervous system occurs in approximately 40% of patients and is being eliminated in 70% of cases^10^. The use of drugs in inadequate doses and intervals, and the lack of clinical monitoring are two of the main causes of ANS in patients with HIV and syphilis co-infection.

In our study, 73 (21%) patients with HIV and *T. pallidum* coinfection antecedents at some point of the HIV infection, indicating that regional behavioral factors can enhance the patient’s vulnerability. There has been an increase in PVHIV cases worldwide^12^ ^,^ ^13^, as well as of syphilis, and this has influenced the increase of co-infection, with prevalence varying between approximately 9.5%^5^. Of the 73 patients with treponemic test reagent for syphilis, 42.5% were considered cured after treatment and adequate follow-up with negative VDRL. We also observed that, among the 33 patients with treponemic and non-treponemic reagent tests, low income, fertile age, male sex, low schooling, and multiple partners were factors that collaborated with co-infection. These findings are compatible with the HIV epidemiological data in the country^13^, which indicates that this disease, despite seemingly declining in the general population, still increases in these groups. Lack of condom use is also significantly associated with HIV and other STIs^14^.

With regard to the clinical-epidemiological characteristics, of the 19 patients assessed for ANS, having less than 21 sex partners in life, adequate previous treatment, stable relationship, and heterosexuality showed a low frequency in the development of ANS (Table 2), which can be expected due to the low global prevalence of syphilis (0,5%) and HIV (0,8%)^15^ ^,^ ^16^. The mean prevalence of syphilis among sex workers and men who have sex with men is 3.2% and 6%, respectively, which confirms that these behavioral characteristics actually contribute to the development of the disease^15^. 

**TABLE 2:** Distribution of the neurosyphilis among the 19 patients with HIV/syphilis co-infection, Cornélio Procópio, State of Paraná, between 10/01/2018 and 09/30/2019.

	Neurosyphilis		
	No	Yes	Total(%)
Characteristics	n(%)	n(%)	
**Marital status**			
Married/Cohabiting	**6(55)**	**3(38)**	9(47)
Other	**5(45)**	**5(62)**	10(53)
Total	11(58)	8(42)	19(100)
**Exposure**			
Heterosexual	**6(55)**	**3(38)**	9(47)
Other	**5(45)**	**5(62)**	10(53)
Total	11(58)	8(42)	19(100)
**Partners**			
<21	**5(45)**	**3(38)**	8(42)
>21	**6(55)**	**5(62)**	11(58)
Total	11(58)	8(42)	19(100)
**Licit drugs**			
Non-users	**4(36)**	**4(50)**	8(42)
Users	**7(64)**	**4(50)**	11(58)
Total	11(58)	8(42)	19(100)
**Illicit drugs**			
Non-users	**10(91)**	**6(75)**	16(84)
Users	**1(09)**	**2(25)**	3(16)
Total	11(58)	8(42)	19(100)
**Previous treatment**			
Inadequate, or without history of previous treatment	**9(82)**	**7(88)**	16(84)
Adequate	**2(18)**	**1(12)**	3(16)
**Total**	**11(58)**	**8(42)**	**19(100)**

n: number of patients.

In our study, we verified that the use of illicit drugs is an important factor for HIV and syphilis co-infection, enhancing the chance of neurosyphilis. In a Brazilian study, it was demonstrated that the odds ratio of HIV infection is higher for people exposed to sexual violence at least once in their lifetimes and are under the use of illicit drugs at least once a week, especially crack cocaine. The same study showed that HIV infection was higher in those who presented a reagent treponemic test for syphilis^14^.

In the studied population, we verified that 26.3% of the patients with ANS did not contemplate the national protocol indications for CSF collection at the time of the study. This result indicates a diagnosis loss of neurosyphilis among patients with HIV infection, revealing the fragility of follow-up and vigilance, resulting in the morbidity and mortality of co-infection.

Considering the high patient turnover in reference services, the absence of consultations, frequent treatment abandonment, monitoring, and follow-up are challenging. Moreover, the complexity of the lumbar puncture technique for CSF collection is an obstacle to its realization. In addition, due to frequent domicile changes, many patients do not attend medical appointments with detailed information on syphilis treatment and laboratory monitoring carried out in other services, contributing to a delay in ANS investigation, such as what occurred to patients in this study. 

The high prevalence of co-infection at the time of HIV diagnosis was found in our study, and the current epidemic situation of syphilis in Brazil reinforces the necessity of collecting routine outpatient laboratory examinations for the diagnosis of asymptomatic infections.

In individuals with HIV infection, serum VDRL titers > 1:32 are predictive of neurosyphilis in all individuals with syphilis, while CD4LT count < 350 cells/mm^3^ is an additional risk factor for neurosyphilis^8^. However, the VDRL test used to assess the CSF sample has a sensitivity variability of 30 to 70%^10^.

In our study, we found a 2.3% ANS frequency among patients followed up with SAE. Rotman *et al.* (2019) retrospectively studied 230 patients with CSF analysis for 10 years in American hospitals and found 70 (8.57%) ANS cases^4^. However, we highlight that each country’s context is different, and clinical-epidemiological and social-demographical characteristics are determinants in the development of neurosyphilis.

Lack of outpatient follow-up and examinations in some patients limited CSF collection, resulting in a smaller sample size. Since neurosyphilis notification is not compulsory in Brazil, this hampers a comparative analysis of national data, and does not allow the performance of a historic line and comparison with other comorbidities.

In conclusion, the frequency of ANS found in our study was low, and a significant proportion of these patients did not meet the criteria for lumbar puncture. When we analyzed the patients with a previous history of syphilis treatment, the findings indicated that patients that were adequately treated had a good protective response to neurosyphilis. Thus, clinical-epidemiological and social-demographical variables as predictive factors of ANS should be considered. In addition, detailed information of previous treatment and laboratory follow-up within and between institutions is fundamental for the timely investigation of each case and indication of CSF collection.
