# Supplementary material for: Asymptomatic Neurosyphilis in HIV infected patients at a Brazilian HIV and AIDS specialized service: a cross sectional study
Source: Rev Soc Bras Med Trop. 2022 Feb 25;55:e0418-2021. doi: 10.1590/0037-8682-0418-2021 (PMC8909440; doi:10.1590/0037-8682-0418-2021)
Supplement: Supplementary file 1 [file 1678-9849-rsbmt-55-e0418-2021-supp1.pdf]

SUPPLEMENTARY TABLE 1: General characteristics of the 19 HIV/syphilis co-infected patients that collected CSF, in Cornélio Procópio, State of Paraná, between 10/01/2018 and 09/30/2019.

Pacient	Previous syphilis treatment	Viral load (copies/mL)	CD4LT (cells/mm^3)	VDRL (serum)	VDRL (CSF)	Leukocytes (CSF) (leuk./mm^3)	Proteins (CSF) (mg/dL)	Red cells (CSF) (cells/ mm^3)	ANS	CPTG'S critéria for CSF collection
**1**	IT	90	452	1/4	NR	3	31.1	0	No	No
**2**	AT	ND	260	1/8	NR	5	9	12	No	**Yes**
**3**	IT	<min lim	1,090	1/16	NR	3	10.8	165	No	No
**4**	NT	17,705	217	1/16	NR	4	**46.7**	**10**	**Yes**	**Yes**
**5**	IT	ND	449	1/4	NR	8	**61**	**14**	**Yes**	No
**6**	IT	ND	565	1/2	NR	2	**48.5**	**73**	**Yes**	No
**7**	NT	ND	120	1/32	NR	**38**	**175.3**	**0**	**Yes**	**Yes**
**8**	IT	ND	798	1/16	NR	2	11.1	0	No	No
**9**	AT	25,458	208	1/64	**1/1**	13	**46.5**	**0**	**Yes**	**Yes**
**10**	AT	<min lim	589	1/8	NR	1	12.4	4	No	No
**11**	NT	<min lim	283	1/4	NR	5	33.7	7	No	**Yes**
**12**	NT	ND	1021	1/4	NR	1	30.2	5	No	No
**13**	IT	<min lim	1244	1/8	NR	2	17.8	0	No	No
**14**	AT	ND	412	1/16	NR	2	12.5	0	No	No
**15**	IT	ND	390	1/2	NR	6	**49.9**	**1**	**Yes**	No
**16**	IT	ND	492	1/2	NR	1	17	0	No	No
**17**	IT	ND	537	1/2	NR	**85**	35	2	**Yes**	No
**18**	IT	<min lim	409	1/4	NR	0	**40.7**	**1**	**Yes**	No
**19**	IT	261	897	1/4	NR	3	30.9	126	No	No

HIV: human immunodeficiency virus; **AT:** adequate previous treatment and laboratorial monitoring for syphilis; **IT:** inadequate previous treatment and laboratorial monitoring for syphilis; **NT:** no syphilis treatment before the research; **CD4LT:** CD4+ T lymphocytes; **VDRL:** Veneral Disease Research Laboratory; **CSF:** cerebrospinal fluid; **ND:** not detectable; **<min lim:** lower than the minimum detection limit; **NR:** not reagent; **ANS:** asymptomatic neurosyphilis; **CPTG:** Clinical Protocol and Therapeutic Guidelines.
